# Supplementary material for: Neutralization of hepatitis B virus with vaccine-escape mutations by hepatitis B vaccine with large-HBs antigen
Source: Nat Commun. 2022 Sep 5;13:5207. doi: 10.1038/s41467-022-32910-z (PMC9441830; doi:10.1038/s41467-022-32910-z)
Supplement: Supplementary file 1 — Supplementary Information [file 41467_2022_32910_MOESM1_ESM.pdf]

Supplementary Information

Neutralization of Hepatitis B Virus with Vaccine-Escape Mutations by Hepatitis B Vaccine with Large-HBs Antigen

Ayaka Washizaki, Asako Murayama, Megumi Murata, Tomoko Kiyohara, Keigo Yato, Norie Yamada, Hussein Hassan Aly, Tomohisa Tanaka, Kohji Moriishi, Hironori Nishitsuji, Kunitada Shimotohno, Yosumasa Goh, Ken J. Ishii, Hiroshi Yotsuyanagi, Masamichi Muramatsu, Koji Ishii, Yoshimasa Takahashi, Ryosuke Suzuki, Hirofumi Akari, Takanobu Kato.

Supplementary Fig. 1

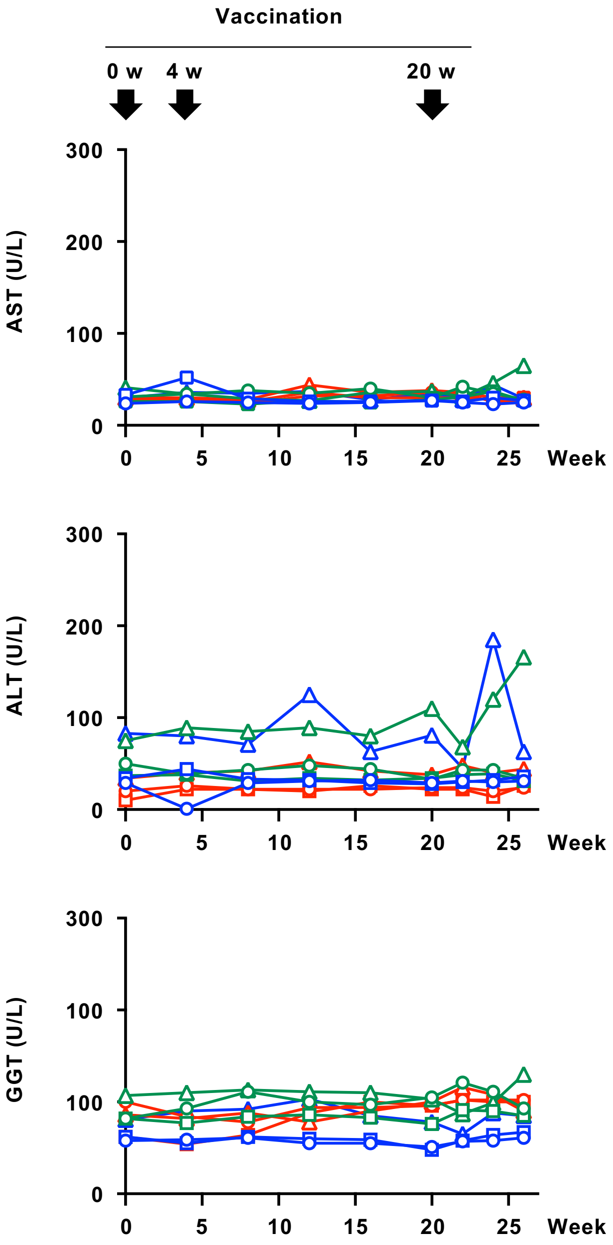

Supplementary Fig. 1. Monitoring of liver enzyme levels during immunization.
The liver function of rhesus macaques immunized with the S-HBs vaccine (blue), L-HBs+K3-SPG (green), or L-HBs+Addavax (red) was monitored by measuring alanine aminotransferase (ALT), aspartate aminotransferase (AST), and gamma-glutamyl transpeptidase (GGT) levels in serum.

Supplementary Fig. 2

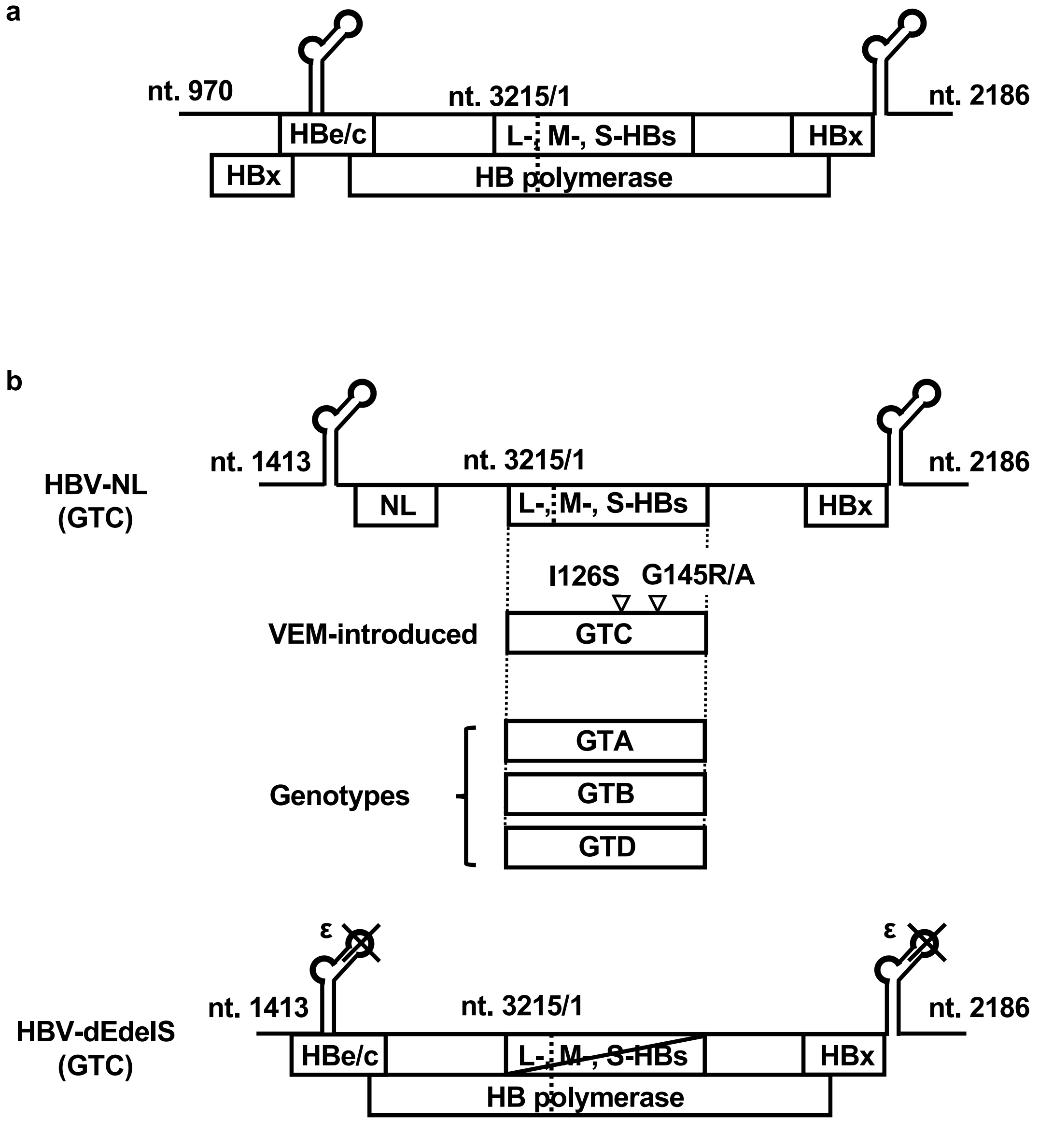

Supplementary Fig. 2. Structure of plasmids used to generate HBVcc and HBV/NL in this study.

(a) The structure of the plasmid used to generate HBVcc. This construct contains a 1.38-fold HBV genome (4,432 bp) of the HBV GTC strain and is capable of generating pregenomic RNA and expressing all HBV proteins. (b) The structure of plasmids for the HBV reporter virus HBV/NL. The constructs of the encapsidation-competent reporter plasmid (HBV-NL) and the encapsidation-incompetent plasmid (HBV-dEdelS) were prepared. The expression of HBsAg from HBV-dEdelS was eliminated by modification of the start codons of all ORFs of HBsAg species. The VEM-introduced HBV-NL plasmids were generated by introducing VEMs of I126S and G145R/A in the S-HBs region of the HBV-NL plasmid. The chimeric HBV-NL plasmids were generated by replacing the L-HBs region with those of the GTA, GTB, and GTD strains. NL; NanoLuc luciferase.

Supplementary Fig. 3

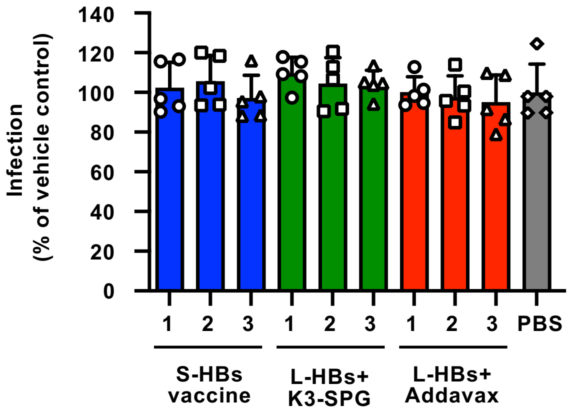

Supplementary Fig. 3. Neutralizing activities of preimmune serum.

The neutralizing activities of preimmune serum samples were assessed in the HBV/NL-WT infection system. The cell culture-generated HBV/NL-WT (GTC, 20 GEq/cell) was mixed with preimmune serum samples of rhesus macaques immunized with S-HBs vaccine (blue), L-HBs+K3-SPG (green), or L-HBs+Addavax (red) for 1 h at 37° C and infected into G2/NT18-C cells. The infection efficiencies were assessed by measuring the NL activities in 5 wells of infected cells at 7 days after infection and compared with the vehicle (PBS)-treated control. The mean ± SD is indicated.

Supplementary Fig. 4

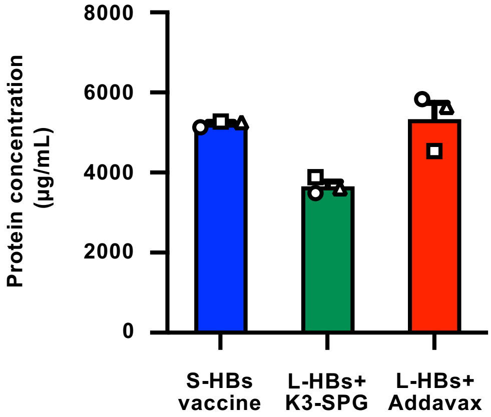

Supplementary Fig. 4. Protein concentrations of purified antibodies.
The induced antibodies were purified from the plasma samples of Bimmugen- (blue), L-HBs+K3-SPG- (green), and L-HBs+Addavax- (red) immunized rhesus macaques, and the protein concentrations were measured by the BCA protein assay. The mean ± SE of three animals is indicated.

Supplementary Fig. 5

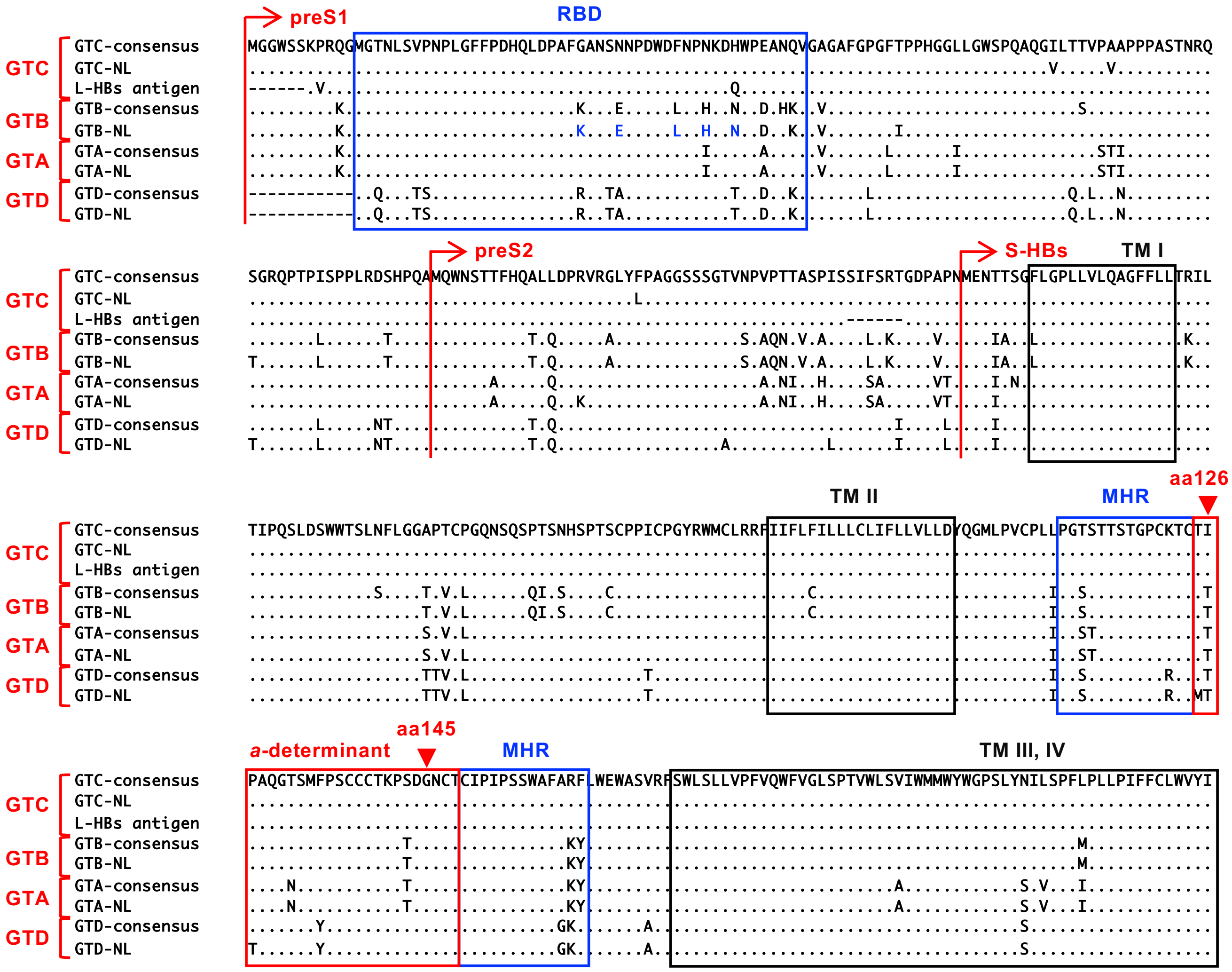

Supplementary Fig. 5. Alignment of amino acid sequences in the preS1-HBs regions of the HBV strains GTA, GTB, GTC, and GTD.

The consensus amino acids of HBV strains of GTC, GTB, GTA, and GTD were retrieved from the Hepatitis Virus Database (http://s2as02.genes.nig.ac.jp/db/) and aligned with the HBV strains used in this study. An identical amino acid is indicated by a dot and a deletion by a dash. GTC-NL, GTB-NL, GTA-NL, and GTD-NL indicate the clones used in HBV/NL of each genotype. L-HBs antigen indicates the amino acid sequence of the antigen used for L-HBsAg provided by Beacle Inc. (Kyoto, Japan). The inverted red triangle indicates the position of the VEMs assessed in this study. The GTB-specific amino acid residues in the RBD are indicated in blue. RBD, receptor-binding domain; MHR, major hydrophilic region; TM, transmembrane segment.

Supplementary Fig. 6

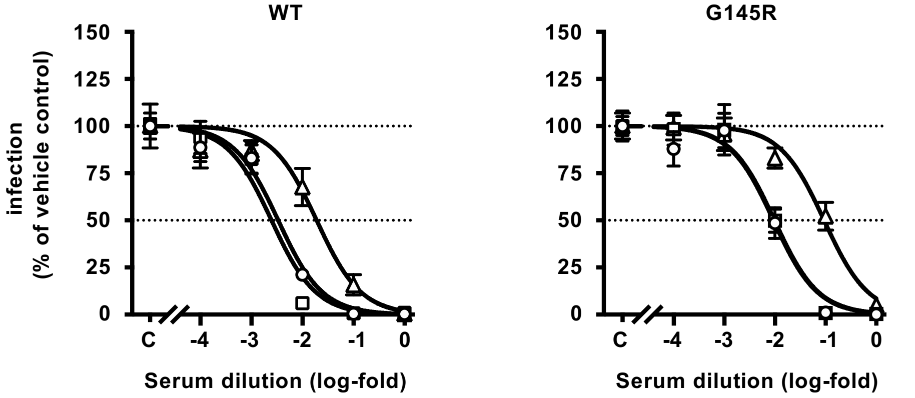

Individual	WT		G145R		Fold
	IC50<br>(fold dilution)	95%CI<br>(fold dilution)	IC50<br>(fold dilution)	95%CI<br>(fold dilution)	
1	3.35×10^{-3}	$2.09 \times 10^{-3} – 5.36 \times 10^{-3}$	9.32×10^{-3}	$5.05 \times 10^{-3} – 1.71 \times 10^{-2}$	2.78
2	2.48×10^{-3}	$1.25 \times 10^{-3} – 4.93 \times 10^{-3}$	9.82×10^{-3}	$6.39 \times 10^{-3} – 1.51 \times 10^{-2}$	3.96
3	1.90×10^{-2}	$9.95 \times 10^{-3} – 3.68 \times 10^{-2}$	9.36×10^{-2}	$6.16 \times 10^{-2} – 1.41 \times 10^{-1}$	4.93

Supplementary Fig. 6. Neutralizing activities of S-HBs vaccine-induced antibodies in humans against HBV with VEM.

The neutralizing activities of S-HBs vaccine-induced antibodies in humans to HBV/NL-WT and HBV/NL-G145R were evaluated. Serum samples were obtained from 3 individuals immunized with the S-HBs vaccines and antibodies were purified. HBV/NL-WT or -G145R (20 GEq/cell) was mixed with serially diluted antibodies for 1 h at 37˚C and was used to infect G2/NT18-C cells. The infection efficiencies at the indicated dilutions of antibodies were assessed by measuring the NL activities in infected cells in 5 wells at 7 days after infection and the mean ± SD was indicated at each dilution. The dose-response curves were drawn with the log (inhibitor) vs. normalized response model, and the IC_{50} values were calculated. The calculated IC_{50} values and the fold IC_{50} value of HBV/NL-G145R in relation to that of HBV/NL-WT are indicated in the lower panel.

Supplementary Fig. 7

a

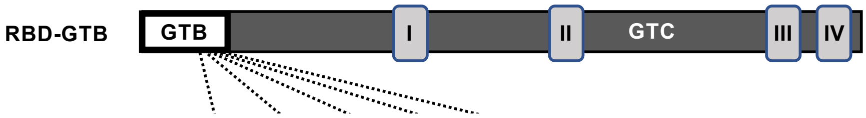

Position	35	39	45	48	51
GTB	K	E	L	H	N
GTC	G	N	F	N	H

b

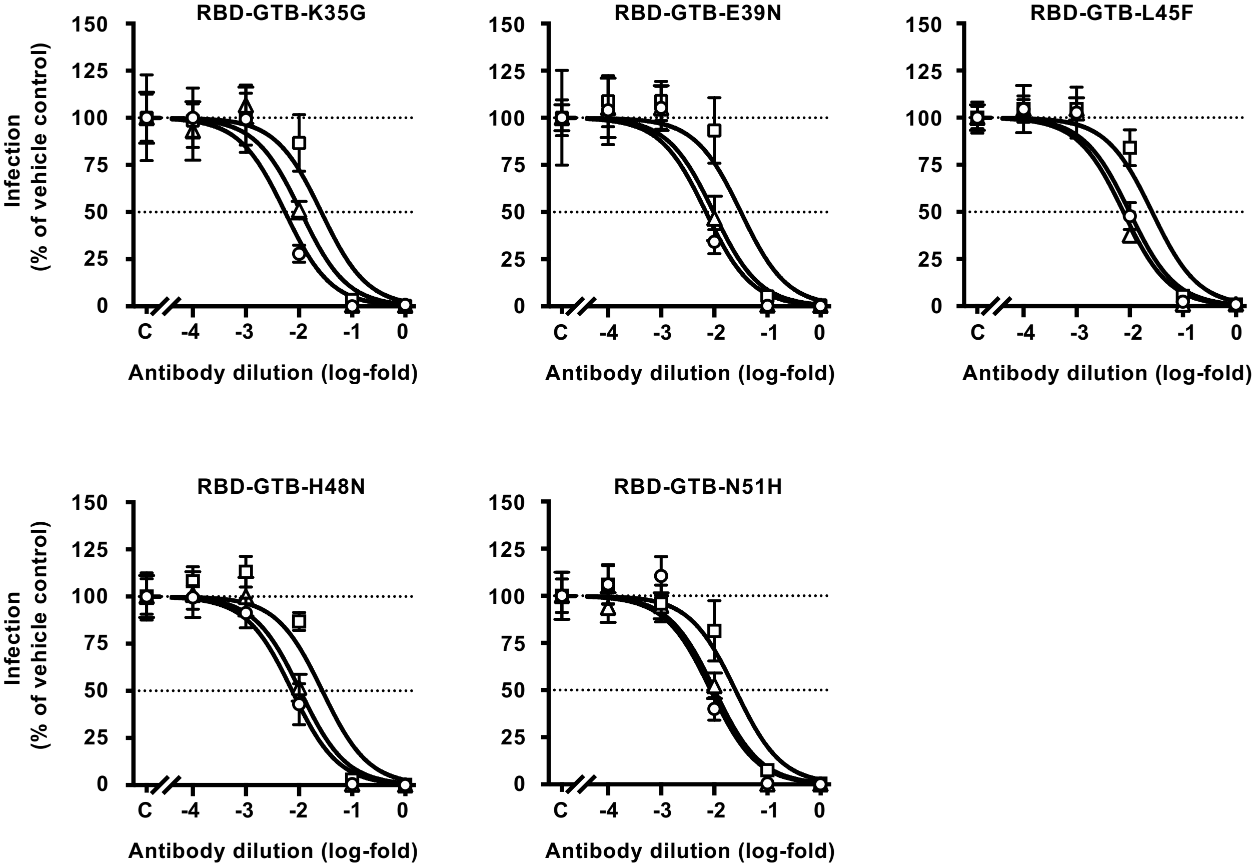

c

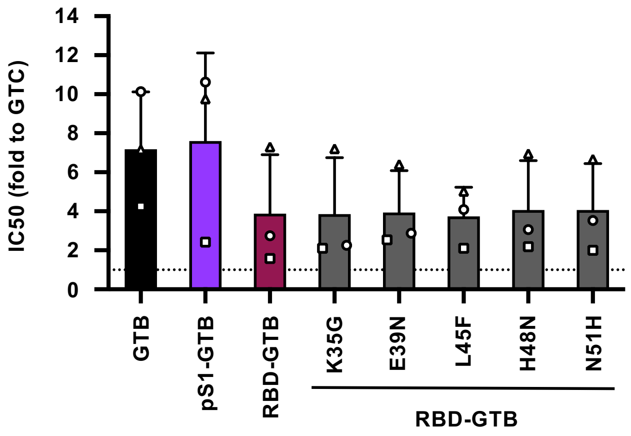

Supplementary Fig. 7. Amino acids responsible for attenuated neutralizing activities to GTB. (a) There are five GTB-specific amino acid residues in the RBD (Supplementary Fig. 5). To identify the responsible amino acid among them, one of these amino acids in HBV/NL-RBD-GTB was changed to the GTC type, and the neutralizing activities were evaluated by L-HBs+Addavax-induced antibodies. (b) The neutralizing activities of L-HBs+Addavax-induced antibodies to HBV/NL-RBD-GTB-K35G, -E39N, -L45F, -H48N, and -N51H were evaluated. These reporter viruses were mixed with serially diluted antibodies of rhesus macaques immunized with L-HBs+Addavax for 1 h at 37˚C and used to infect G2/NT18-C cells. The infection efficiencies were assessed by measuring the NL activities of infected cells in 5 wells at 7 days after infection. (c) The IC50 values were calculated by drawing the dose-response curves with the log (inhibitor) vs. normalized response model and indicated the folds to that of HBV/NL-WT (GTC). The mean ± SD of three animals is indicated.

Supplementary Table 1.
IC_{50} values of HB vaccine-induced antibodies to HBV with VEMs.

Macaque	Virus	IC_{50} (fold dilution)	95%CI (fold dilution)	Ratio (fold of WT)
S-HBs vaccine 1	WT	3.34×10^{-3}	1.79×10^{-3} – 6.23×10^{-3}	-
	I126S	3.97×10^{-3}	3.10×10^{-3} – 5.08×10^{-3}	1.19
	G145R	3.51×10^{-2}	1.78×10^{-2} – 6.92×10^{-2}	10.5
	G145A	1.18×10^{-2}	7.28×10^{-3} – 1.90×10^{-2}	3.53
S-HBs vaccine 2	WT	1.82×10^{-2}	9.80×10^{-3} – 3.37×10^{-2}	-
	I126S	2.21×10^{-2}	1.12×10^{-2} – 4.36×10^{-2}	1.22
	G145R	1.26×10^{-1}	6.41×10^{-2} – 2.55×10^{-1}	6.96
	G145A	6.05×10^{-2}	3.31×10^{-2} – 1.10×10^{-1}	3.33
S-HBs vaccine 3	WT	8.90×10^{-3}	6.02×10^{-3} – 1.31×10^{-2}	-
	I126S	1.04×10^{-2}	6.93×10^{-3} – 1.55×10^{-2}	1.17
	G145R	7.00×10^{-2}	3.89×10^{-2} – 1.25×10^{-1}	7.86
	G145A	2.51×10^{-2}	1.30×10^{-2} – 5.41×10^{-2}	2.82
L-HBs+Addavax 1	WT	2.43×10^{-3}	1.07×10^{-3} – 5.53×10^{-3}	-
	I126S	2.28×10^{-3}	1.17×10^{-3} – 4.45×10^{-3}	0.939
	G145R	1.81×10^{-3}	1.05×10^{-3} – 3.11×10^{-3}	0.745
	G145A	2.18×10^{-3}	1.21×10^{-3} – 5.47×10^{-3}	0.897
L-HBs+Addavax 2	WT	1.26×10^{-2}	7.30×10^{-3} – 2.16×10^{-2}	-
	I126S	1.17×10^{-2}	7.86×10^{-3} – 1.75×10^{-2}	0.935
	G145R	1.30×10^{-2}	4.88×10^{-3} – 3.29×10^{-2}	1.03
	G145A	1.38×10^{-2}	1.01×10^{-2} – 2.03×10^{-2}	1.10
L-HBs+Addavax 3	WT	1.52×10^{-3}	9.06×10^{-4} – 2.55×10^{-3}	-
	I126S	1.43×10^{-3}	8.17×10^{-4} – 2.51×10^{-3}	0.946
	G145R	1.49×10^{-3}	8.71×10^{-4} – 2.57×10^{-3}	0.982
	G145A	1.49×10^{-3}	1.06×10^{-3} – 3.35×10^{-3}	0.981

Supplementary Table 2.
IC_{50} values of HB vaccine-induced antibodies to strains of multiple genotypes.

Macaque	Virus	IC_{50} (fold dilution)	95%CI (fold dilution)	Ratio (fold of GTC)
S-HBs vaccine 1	GTC	3.34×10^{-3}	1.79×10^{-3} – 6.23×10^{-3}	-
	GTA	5.14×10^{-3}	3.52×10^{-3} – 7.43×10^{-3}	1.54
	GTB	4.50×10^{-3}	2.50×10^{-3} – 8.04×10^{-3}	1.35
	GTD	5.65×10^{-3}	3.69×10^{-3} – 8.56×10^{-3}	1.69
S-HBs vaccine 2	GTC	1.82×10^{-2}	9.80×10^{-3} – 3.37×10^{-2}	-
	GTA	2.06×10^{-2}	8.64×10^{-3} – 4.86×10^{-2}	1.14
	GTB	2.13×10^{-2}	1.01×10^{-2} – 4.50×10^{-2}	1.17
	GTD	4.01×10^{-2}	1.59×10^{-2} – 1.02×10^{-1}	2.21
S-HBs vaccine 3	GTC	8.90×10^{-3}	6.02×10^{-3} – 1.31×10^{-2}	-
	GTA	1.11×10^{-2}	6.31×10^{-3} – 1.94×10^{-2}	1.25
	GTB	1.11×10^{-2}	5.34×10^{-3} – 2.22×10^{-2}	1.24
	GTD	2.38×10^{-2}	1.10×10^{-2} – 5.15×10^{-2}	2.67
L-HBs+Addavax 1	GTC	2.43×10^{-3}	1.07×10^{-3} – 5.53×10^{-3}	-
	GTA	2.70×10^{-3}	1.39×10^{-3} – 5.27×10^{-3}	1.11
	GTB	2.46×10^{-2}	8.74×10^{-3} – 6.89×10^{-2}	10.1
	GTD	4.17×10^{-3}	2.24×10^{-3} – 7.73×10^{-3}	1.72
L-HBs+Addavax 2	GTC	1.26×10^{-2}	7.30×10^{-3} – 2.16×10^{-2}	-
	GTA	1.66×10^{-2}	9.83×10^{-3} – 2.84×10^{-2}	1.32
	GTB	5.33×10^{-2}	3.59×10^{-2} – 7.82×10^{-2}	4.25
	GTD	2.06×10^{-2}	1.26×10^{-2} – 3.40×10^{-2}	1.64
L-HBs+Addavax 3	GTC	1.52×10^{-3}	9.06×10^{-4} – 2.55×10^{-3}	-
	GTA	1.96×10^{-3}	6.01×10^{-4} – 6.32×10^{-3}	1.30
	GTB	1.09×10^{-2}	5.48×10^{-3} – 2.09×10^{-2}	7.16
	GTD	2.31×10^{-3}	1.18×10^{-3} – 4.56×10^{-3}	1.53
