# Supplementary material for: Neutralization of hepatitis B virus with vaccine-escape mutations by hepatitis B vaccine with large-HBs antigen
Source: Nat Commun. 2022 Sep 5;13:5207. doi: 10.1038/s41467-022-32910-z (PMC9441830; doi:10.1038/s41467-022-32910-z)
Supplement: Supplementary file 3 — Reporting Summary [file 41467_2022_32910_MOESM3_ESM.pdf]

nature portfolio

Corresponding author(s): Takanobu Kato

Last updated by author(s): Aug 18, 2022

Reporting Summary

Nature Portfolio wishes to improve the reproducibility of the work that we publish. This form provides structure for consistency and transparency in reporting. For further information on Nature Portfolio policies, see our Editorial Policies and the Editorial Policy Checklist.

Statistics

For all statistical analyses, confirm that the following items are present in the figure legend, table legend, main text, or Methods section.

n/a	Confirmed	
☐	☒	The exact sample size (*n*) for each experimental group/condition, given as a discrete number and unit of measurement
☐	☒	A statement on whether measurements were taken from distinct samples or whether the same sample was measured repeatedly
☐	☒	The statistical test(s) used AND whether they are one- or two-sided<br>*Only common tests should be described solely by name; describe more complex techniques in the Methods section.*
☒	☐	A description of all covariates tested
☒	☐	A description of any assumptions or corrections, such as tests of normality and adjustment for multiple comparisons
☐	☒	A full description of the statistical parameters including central tendency (e.g. means) or other basic estimates (e.g. regression coefficient) AND variation (e.g. standard deviation) or associated estimates of uncertainty (e.g. confidence intervals)
☐	☒	For null hypothesis testing, the test statistic (e.g. *F*, *t*, *r*) with confidence intervals, effect sizes, degrees of freedom and *P* value noted<br>*Give P values as exact values whenever suitable.*
☒	☐	For Bayesian analysis, information on the choice of priors and Markov chain Monte Carlo settings
☒	☐	For hierarchical and complex designs, identification of the appropriate level for tests and full reporting of outcomes
☒	☐	Estimates of effect sizes (e.g. Cohen's *d*, Pearson's *r*), indicating how they were calculated

Our web collection on statistics for biologists contains articles on many of the points above.

Software and code

Policy information about availability of computer code

Data collection	The images of HBcAg positive cells were captured with a BZ-X710 fluorescence microscope (Keyence, Osaka, Japan), and the area size of staining was quantified with the built-in software BZ-3HA ver. 1.3.0.3 (Keyence).
Data analysis	Statistical analysis was performed with GraphPad PRISM 8 software (GraphPad Software, La Jolla, CA).

For manuscripts utilizing custom algorithms or software that are central to the research but not yet described in published literature, software must be made available to editors and reviewers. We strongly encourage code deposition in a community repository (e.g. GitHub). See the Nature Portfolio guidelines for submitting code & software for further information.

Data

Policy information about availability of data

All manuscripts must include a data availability statement. This statement should provide the following information, where applicable:

- Accession codes, unique identifiers, or web links for publicly available datasets
- A description of any restrictions on data availability
- For clinical datasets or third party data, please ensure that the statement adheres to our policy

Source data are provided as a Source Data file.

Field-specific reporting

Please select the one below that is the best fit for your research. If you are not sure, read the appropriate sections before making your selection.

☒ Life sciences ☐ Behavioural & social sciences ☐ Ecological, evolutionary & environmental sciences

For a reference copy of the document with all sections, see nature.com/documents/nr-reporting-summary-flat.pdf

Life sciences study design

All studies must disclose on these points even when the disclosure is negative.

Sample size	- For in vivo experiments, 3 animals were assigned to each vaccinated group. This study is a pilot study using the nonhuman primate. We utilized a small number of animals in each group.<br>- For in vitro experiments, the triplicate assessment was performed in the infection study of cell culture-generated HBV and the quintuplicate assessment was performed in the infection study of HBV reporter viruses.
Data exclusions	No data was excluded.
Replication	All experiments were reproduced at least three times in an independent manner.
Randomization	Animals were randomly assigned which HB vaccines are administered. Other experiments are not available for randomization.
Blinding	Blinding was not done at the vaccination. To detect the crucial side effects of novel vaccines, detailed clinical observations and attention were needed. For the assessment of induced antibodies, the investigator who participated in the studies of neutralization was blinded.

Reporting for specific materials, systems and methods

We require information from authors about some types of materials, experimental systems and methods used in many studies. Here, indicate whether each material, system or method listed is relevant to your study. If you are not sure if a list item applies to your research, read the appropriate section before selecting a response.

Materials & experimental systems

n/a	Involved in the study	
☐	☒	Antibodies
☐	☒	Eukaryotic cell lines
☒	☐	Palaeontology and archaeology
☐	☒	Animals and other organisms
☐	☒	Human research participants
☒	☐	Clinical data
☒	☐	Dual use research of concern

Methods

n/a	Involved in the study	
☒	☐	ChIP-seq
☒	☐	Flow cytometry
☒	☐	MRI-based neuroimaging

Antibodies

Antibodies used	HRP-conjugated anti-human IgG, 0.2 μg/mL, catalog number;P0214, Dako Cytomation.<br>Anti-Hepatitis B-core IgG fraction (polyclonal), 1 μg/mL, catalog number; HBP-023-9, lot number; GZ201510, AUSTRAL Biologicals.<br>Alexa Fluor 555-conjugated anti-rabbit IgG, 1mg/mL, catalog number; A32732, Thermo Fisher Scientific.
Validation	The antibodies used in this study are commercially available and validated by their respective manufacturers.<br>HRP-conjugated anti-human IgG: https://www.agilent.com/store/ja_JP/Prod-P021402-2/P021402-2.<br>Anti-Hepatitis B-core IgG fraction (polyclonal): http://www.australbiologicals.com/index.php?what=catalog&id=68.<br>Alexa Fluor 555-conjugated anti-rabbit IgG: https://www.thermofisher.com/antibody/product/Goat-anti-Rabbit-IgG-H-L-Highly-Cross-Adsorbed-Secondary-Antibody-Polyclonal/A32732.

Eukaryotic cell lines

Policy information about cell lines

Cell line source(s)	HepG2 cell (ECACC), G2/NT18-C (Yamanashi Univ.), HepG2-NTCPsec+ (Institut Pasteur Korea)
Authentication	None of cell lines were authenticated.
Mycoplasma contamination	All cell lines were confirmed negative for mycoplasma contamination.

Commonly misidentified lines (See ICLAC register)	None.

Animals and other organisms

Policy information about studies involving animals; ARRIVE guidelines recommended for reporting animal research

Laboratory animals	Rhesus macaque (3-5 years of age, male or female, approximately 5 kg body weight)
Wild animals	No wild animals were used in this study.
Field-collected samples	This study did not use field-collected samples.
Ethics oversight	The animal experiments were conducted using protocols and experimental procedures that were approved by the Animal Welfare and Animal Care Committee of Kyoto University and were carried out in accordance with the Guidelines for Care and Use of Nonhuman Primates (Version 3) by the Animal Welfare and Animal Care Committee of Kyoto University.

Note that full information on the approval of the study protocol must also be provided in the manuscript.

Human research participants

Policy information about studies involving human research participants

Population characteristics	Serum samples were obtained from three individuals (37-42 years of age, 1 male and 2 female) who were vaccinated with the commercially available S-HBs vaccine in Japan.
Recruitment	Serum samples were obtained from volunteers with written informed consent. Calls for participation in the study were made via posters, e-mails, and announcements. Any biases will not be discussed because the human study in this paper was performed to validate the reliability of the system used.
Ethics oversight	The experiment was approved by the Ethics Committees of National Institute of Infectious Diseases (approval number; 780).

Note that full information on the approval of the study protocol must also be provided in the manuscript.
